# Exposure, respiratory symptoms, lung function and inflammation response of road-paving asphalt workers

**DOI:** 10.1136/oemed-2017-104983

**Published:** 2018-05-30

**Authors:** Yiyi Xu, Monica Kåredal, Jörn Nielsen, Mariana Adlercreutz, Ulf Bergendorf, Bo Strandberg, Ann-Beth Antonsson, Håkan Tinnerberg, Maria Albin

**Affiliations:** 1 Division of Occupational and Environmental Medicine, Laboratory Medicine, Lund University, Lund, Sweden; 2 Section of Occupational and Environmental Medicine, Institute of Medicine, Sahlgrenska Academy at the University of Gothenburg, Gothenburg, Sweden; 3 IVL Swedish Environmental Research Institute, Stockholm, Sweden; 4 Unit of Occupational Medicine, Institute of Environmental Medicine, Karolinska Institutet, Stockholm, Sweden

**Keywords:** occupational exposure, asphalt, rubber asphalt, respiratory symptoms, lung function test, interleukin, C-reactive protein

## Abstract

**Background:**

Controversy exists as to the health effects of exposure to asphalt and crumb rubber modified (CRM) asphalt, which contains recycled rubber tyres.

**Objective:**

To assess exposures and effects on airway symptoms, lung function and inflammation biomarkers in conventional and CRM asphalt road pavers.

**Methods:**

116 conventional asphalt workers, 51 CRM asphalt workers and 100 controls were investigated. A repeated-measures analysis included 31 workers paving with both types of asphalt. Exposure to dust, nitrosamines, benzothiazole and polycyclic aromatic hydrocarbon (PAH) was measured in worksites. Self-reported symptoms, spirometry test and blood sampling were conducted prework and postwork. Symptoms were further collected during off-season for asphalt paving.

**Results:**

Dust, PAHs and nitrosamine exposure was highly varied, without difference between conventional and CRM asphalt workers. Benzothiazole was higher in CRM asphalt workers (p<0.001). Higher proportions of asphalt workers than controls reported eye symptoms with onset in the current job. Decreased lung function from preworking to postworking was found in CRM asphalt workers and controls. Preworking interleukin-8 was higher in CRM asphalt workers than in the controls, followed by a decrement after 4 days of working. No differences in any studied effects were found between conventional and CRM asphalt paving.

**Conclusion:**

CRM asphalt workers are exposed to higher benzothiazole. Further studies are needed to identify the source of nitrosamines in conventional asphalt. Mild decrease in lung function in CRM asphalt workers and work-related eye symptoms in both asphalt workers were observed. However, our study did not find strong evidence for severe respiratory symptoms and inflammation response among asphalt workers.

**Key messages**

**What is already known about this subject?**

- Exposure and health effects of emissions from asphalt and crumb rubber modified (CRM) asphalt paving are not clear.

**What are the new findings?**

- Nitrosamines were also found in conventional asphalt, suggesting sources other than reused rubber tyres may contribute to exposure.
- Decreased lung function after 4 days of paving in CRM asphalt workers and more reports of work-related eye symptoms in both conventional and CRM asphalt workers were observed.

**How might this impact on policy or clinical practice in the foreseeable future?**

- Future research to identify the source of nitrosamines in asphalt paving is needed to eliminate (if possible) the source and to guide risk assessment for asphalt paving.

## Introduction

Possible adverse health effects of exposure to asphalt fumes have been a subject of discussion for several years. Studies have shown that asphalt road pavers are more likely to acquire eye and upper airway symptoms,^1 2^ and experience lung function decline^3–5^ and mild inflammation response.^6 7^ However, data on current exposure conditions for risk assessment are limited, which in turn bring difficulties in setting relevant occupational exposure limits (OELs). Recently, the Swedish Criteria Group for Occupational Standards assessed that there are insufficient data to determine the critical effects of exposure to asphalt fumes during road paving.^8^

Moreover, the health impacts of exposure to crumb rubber modified (CRM) asphalt have come to the fore of the debate with the increasing use of crumbs from reused rubber tyres mixed into asphalt. However, the emissions during paving operation on site have not been extensively investigated, with an even limited number of studies focused on potential health risks.^9–12^ The National Institute for Occupational Safety and Health reported higher emissions of polycyclic aromatic hydrocarbon (PAH) and benzothiazole, a mucosal irritant, in CRM asphalt paving, together with two to three times higher prevalence of self-reported eye, nasal and throat irritation as compared with conventional asphalt workers.^9^ But a review pointed out that the effects of CRM asphalt, if any, were relatively small.^11^ Higher PAH and benzothiazole emissions from CRM asphalt were also found in a Swedish report^13^ and were recently confirmed in an experimental setting, in addition to higher particle emissions for CRM as compared with conventional asphalt.^14^

The aims of this study are to assess exposures to asphalt fumes, together with the possible health effects on airway symptoms, lung function and inflammation response in asphalt workers; and further to investigate if paving with CRM asphalt is related to higher exposure and greater adverse effects than with conventional asphalt.

## Materials and methods

### Study participants

From 2012 to 2015, we went to 41 asphalt paving sites and investigated 116 conventional asphalt workers and 51 CRM asphalt workers; all were men. We also recruited 100 male controls who worked with landscaping and gardening outdoors. All asphalt workers were investigated between April and October (road paving season), while around 50% of the controls were investigated between January and March due to availability. The participants were investigated preworking on Monday morning (06:00–08:00) and postworking on Thursday afternoon (12:00–16:00) after four consecutive working days. There were 31 workers who worked with both conventional and CRM asphalt in different years; therefore, they were investigated twice (preworking and postworking) when laying conventional asphalt and twice (preworking and postworking) when laying CRM asphalt. The details of recruitment and investigation were described elsewhere.^15^ All study participants gave informed written consent to take part in the study.

### Personal exposure monitoring

Personal sampling of respirable dust, total dust, total airborne PAHs (particle and gas phase), benzothiazole and nitrosamines was performed in around 25–50 asphalt workers at several worksites on 17 separate days (other than the days for medical investigations). Personal air samplers were worn in the breathing zone by each worker. A stationary sampling close to the worker was carried out only if it was not possible to place air sampler personally due to tight conditions (mainly for the paving machine drivers). Respirable dust was collected on 37 mm mixed cellulose ester (MCE) membrane filters, with a pore size of 0.8 µm (Millipore AAWP03700)  and fitted to cyclones (BGI4L, BGI, USA). The total dust and airborne particulate PAHs were simultaneously collected on 37 mm polytetrafluoroethylene (PTFE) membrane filters (Pall Life Sciences, USA) fitted in conductive filter cassettes. Adsorption tubes (XAD-2, SKC, USA) were used downstream the total dust filters to collect gaseous PAH components and benzothiazole. Nitrosamines were collected on a special type of sampler (Thermosorb/N adsorption tubes). The flow rates were 2.2 L/min for respirable dust, and 1.5 L/min for total dust, total airborne PAHs and nitrosamines, respectively.

The respirable and total dust filters were weighted according to standard procedures. PAHs were analysed according to a previously described procedure.^16^ In short, before extraction, an internal standard mixture containing 16 US Environmental Protection Agency (EPA) priority PAHs were added to the samples. The target compounds were analysed using high-resolution gas chromatography/low-resolution mass spectrometry. The total airborne PAH refers to the sum of 32 PAH components (16 US EPA PAHs and 16 alkylated species).^16^ Benzothiazole was analysed using deuterated benzothiazole as an internal standard. Nine species of nitrosamines (N-nitroso-dimethylamine, N-nitroso-methylethylamine, N-nitroso-pyrrolidin, N-nitroso-diethylamine, N-nitroso-piperidine, N-nitroso-morpholine, N-nitroso-di-N-propylamine, N-nitroso-di-N-butylamine and N-nitroso-phenylamine) were determined by liquid chromatography/tandem mass spectrometry.

### Questionnaire investigation and self-reported symptoms

Three questionnaires were used in the study: one general health questionnaire (preworking questionnaire) was completed before Monday morning, including occupational history, medical and disease history, smoking history (cigarette smoking and smokeless tobacco ‘snus’ (a moist snuff placed under the upper lip) use), together with eye symptoms (redness/secretion/swelling) and nasal symptoms (runny nose/nasal congestion/sneezing), nasal bleeding, and wheezing, chest tightness, shortness of breath and cough in the last week. One shorter questionnaire (postworking questionnaire) was completed on Thursday afternoon, focused on the same symptoms that occurred during the working week. An additional questionnaire (off-season questionnaire) was distributed by mail during the winter season after the onsite investigation and was required to be sent back before the new paving season started, from which information on the same symptoms was collected together with working circumstances during the winter vacation.

### Spirometry test

Forced vital capacity (FVC) and forced expiratory volume in 1 s (FEV_1_) were measured preworking on Monday morning and postworking on Thursday afternoon with a computerised spirometer (Spirare 3, Diagnostica, Oslo, Norway). Each participant repeated the spirometry test for at least three times for test reliability according to the European Respiratory Society guidelines, and the ‘best’ of the three accepted readings was accepted.^17^ Per cent of predicted values of FVC (FVC (% predicted)) and FEV_1_ (FEV_1_ (% predicted)) according to Berglund *et al* 18 were used in the statistical analysis.

### Inflammation biomarkers and Phadiatop allergy test

Peripheral blood was obtained preworking and postworking onsite and transported to the laboratory in a cool bag with ice pack. EDTA plasma and serum were isolated and stored at −80°C until analysis. Plasma C reactive protein (CRP) was measured by immunoturbidimetry at the Department of Clinical Chemistry at Lund University Hospital, according to standard protocols. Serum interleukin-6 (IL-6) and interleukin-8 (IL-8) were measured at the Division of Occupational and Environmental Medicine, Laboratory Medicine, Lund University, using Luminex xMAP Technology on a Bio-Plex 200 platform (Bio-Rad, Hercules, California, USA), according to the instructions from the manufacturer. The results were evaluated in Bio-Plex Manager V.6.0 (Bio-Rad). Four per cent of IL-8 was below the limit of detection (LOD), and the concentrations were replaced with half of LOD before statistical analysis. Seventy-seven per cent of the IL-6 was below the LOD; therefore, it was not analysed further. Phadiatop (allergy screening) test was analysed by fluoroimmunoassay at the Clinical Immunology and Transfusion Medicine in Region Skåne. The constituent of allergen includes animals (dog, cat, horse), pollen (timothy, birch, mugwort), mites (*Dermatophagoides pteronyssinus*, *D farinae*) and moulds (*Cladosporium*).

### Statistical analysis

Self-reported symptoms were grouped into two categories for statistical analysis:

1. Upper airway symptoms: eye symptoms (redness/secretion/swelling), nasal symptoms (runny nose/nasal congestion/sneezing) and nasal bleeding.
2. Lower airway symptoms: wheezing, chest tightness, shortness of breath and cough.

Aggregate categories were defined as the presence of at least one of the respective symptoms in the group (dichotomous ‘positive/negative’). The asymptotic McNemar test was adopted to test the differences in reports of upper or lower airway symptoms between preworking and postworking questionnaires, as well as between preworking and off-season questionnaires, in each occupational group. Then, newly developed symptoms during the working week (ie, participants did not report such symptoms on the preworking questionnaire, but reported those symptoms on the postworking questionnaire) were calculated for comparison across three occupational groups. Logistic regression was adopted to estimate the ORs in the two asphalt groups compared with the controls. For analysis of spirometry test and inflammation biomarkers, the absolute changes (Δ) from preworking to postworking measurements were calculated for each participant. General linear regression was performed to assess the associations between different outcomes and various exposure indexes (occupational groups, years working as an asphalt worker). Interaction between allergy (positive or negative) and occupational groups was tested, but no interaction was found. Age, smoking history, cigarette pack-year and allergy were included in the regression models for adjustments since when individually added to regression models the β estimates for exposure indexes changed by more than 10%.

A repeated-measures analysis was performed based on the 31 asphalt workers who worked with both conventional and CRM asphalt in different years to explore if CRM asphalt exposure was related to greater adverse effects. Asymptotic McNemar test was used to analyse the difference in the self-reported symptoms. Linear mixed model was adopted to analyse the difference in the changes of lung function and inflammation biomarkers (Δ) between CRM and conventional asphalt paving. Only baseline values were included in the models for adjustment. All statistical analyses were completed using SPSS V.23.0.

## Results

### Basic characteristics and personal exposures of study participants

The prevalence of current smoking was slightly higher in CRM asphalt workers and controls than in conventional asphalt workers (p=0.066); the prevalence of current ‘snus’ user was higher in the two asphalt groups than in the controls (p=0.014). The Phadiatop test showed similar prevalence of pollen, animals and mould allergy but different prevalence of mite allergy across three occupational groups, with the highest prevalence (24%) in the controls (p=0.049; table 1).

**Table 1:** Characteristics of the study participants in three occupational groups

	Conventional asphalt workers (n=116)	CRM asphalt workers (n=51)	Controls (n=100)	P values*
Age	43 (24–59)	42 (22–61)	46 (24–62)	0.29
BMI	27.5 (22.4–38.2)	27.8 (22.6–34.6)	27.4 (22.2–35.4)	0.89
Years of asphalt work (years)	12 (1–29)	10 (1–38)	–	–
Self-reported COPD (no/yes, %)†	114/0 (0)	44/0 (0)	99/1 (1)	0.90
Diagnosed asthma (no/yes, %)†	106/8 (7)	41/3 (7)	87/13 (13)	0.27
Smoking history (never/ever/current, %)†	84/24/6 (5)	31/5/8 (18)	65/23/12 (12)	0.066
Cigarette pack-year if ever smoker	9 (2–30)	18 (1–95)	14 (1–54)	0.083
Snus history (never/ever/current, %)†	54/15/45 (39)	25/2/17 (39)	66/13/21 (21)	0.014
Phadiatop allergy test (negative/positive, %)‡	72/41 (36)	33/12 (27)	62/34 (35)	0.49
Moulds (negative/positive, %)	111/2 (2)	45/0 (0)	95/1 (1)	0.61
Mites (negative/positive, %)	97/16 (14)	41/4 (9)	73/23 (24)	0.049
Animals (negative/positive, %)	100/13 (12)	43/2 (4)	83/13 (14)	0.27
Pollen (negative/positive, %)	76/37 (33)	35/10 (22)	73/23 (24)	0.25
Grass pollen (negative/positive, %)	80/33 (29)	35/10 (22)	75/21 (22)	0.44
Total IgE‡	30.6 (3.5–291)	30.9 (5.2–281)	31.5 (3.5–279)	0.87

Data presented as median  (5th–95th percentiles) for continuous variables, and count (%) for categorical variables

*P values were derived from one-way analysis of variance for parametric continuous variables, and χ^2^ test or Fisher’s exact test for categorical variables with controls as the comparison group.

†Two conventional asphalt workers and seven CRM asphalt workers did not report disease history and smoking history on the preworking questionnaire.

‡Three conventional asphalt workers, six CRM asphalt workers and four controls did not give consent for blood sampling for allergy test.

BMI, body mass index; COPD, chronic obstructive pulmonary disease; CRM, crumb rubber modified.

Exposure levels of respirable dust, total dust, total airborne PAHs and nitrosamines were highly varied but did not differ between conventional and CRM asphalt workers; however, exposure to benzothiazole was much higher in CRM asphalt workers (table 2). For the nitrosamines, N-nitroso-piperidine was found in all samples and N-nitroso-phenylamine in some of the samples. The highest concentration of total nitrosamine exposure (1.51 µg/m^3^) was observed in one CRM asphalt worker and was above the German technical limit values in the rubber industry (<1 µg/m^3^).^19^

**Table 2:** Exposure concentrations obtained from personal sampling when paving with conventional and CRM asphalt

Exposure parameters	Conventional asphalt		CRM asphalt		P values*
	n	Median (5%–95%)	n	Median (5%–95%)	
Respirable dust (µg/m^3^)	19	240 (10–610)	31	160 (10–1180)	0.73
Total dust (µg/m^3^)	19	180 (10–1180)	18	210 (10–3070)	0.92
Total airborne PAHs (µg/m^3^)†	19	2.75 (0.71–6.24)	18	2.55 (1.32–9.81)	0.23
Benzothiazole (µg/m^3^)	11	0.37 (0.17–2.63)	14	2.09 (1.01–3.69)	<0.001
Nitrosamines (µg/m^3^)‡	15	0.060 (0.020–0.52)	26	0.070 (0.020–1.51)	0.60

*P values were derived from Mann-Whitney test for non-parametric continuous variables.

†Particle and gas phase; sum of 32 PAHs.^18^

‡Sum of 9 nitrosamines.

CRM, crumb rubber modified; PAH, polycyclic aromatic hydrocarbon.

### Self-reported symptoms

The overall response rates to postworking questionnaire and off-season questionnaire were 96% (100% for conventional asphalt workers, 82% for CRM asphalt workers and 98% for controls) and 77% (83% for conventional asphalt workers, 71% for CRM asphalt workers and 73% for controls), respectively. Self-reported preworking and postworking symptoms (table 3) showed no difference in the prevalence of lower or upper airway symptoms in either conventional or CRM asphalt workers. Fewer controls reported lower airway symptoms from postworking than preworking (p=0.079). Conventional asphalt workers showed fewer reports of lower airway symptoms during off-season compared with preworking (ie, during working season) (p=0.077). A similar pattern was, however, also found in the controls (p=0.0074). No difference in newly developed lower or upper airway symptoms was found between two asphalt group and controls. Regarding individual symptoms, eye symptoms (redness/secretion/swelling), nasal symptoms (runny nose/nasal congestion/sneezing) and cough were the most frequently reported airway symptoms in both asphalt workers and controls, without any difference across groups (online supplementary table 1).

10.1136/oemed-2017-104983.supp1 Supplementary data

**Table 3:** Self-reported symptoms from preworking, postworking and off-season questionnaires, and newly developed symptoms during the week in three occupational groups*

Occupational groups	Preworking	Postworking	P values†	Off-season	P values†	Newly developed symptoms		P values‡
	(no/yes, %)	(no/yes, %)		(no/yes, %)		(no/yes, %)	OR (95% CI)‡	
Lower airway symptoms								
Conventional asphalt workers	91/25 (22)	96/20 (17)	0.36	85/11 (11)	0.077	103/13 (11)	0.74 (0.26 to 2.08)	0.56
CRM asphalt workers§	38/9 (19)	33/9 (21)	0.73	32/4 (11)	0.63	33/5 (13)	1.89 (0.54 to 6.66)	0.32
Controls	73/27 (27)	83/15 (15)	0.079	63/10 (14)	0.0074	88/10 (10)	Ref	Ref
Upper airway symptoms								
Conventional asphalt workers	74/42 (36)	70/46 (40)	0.48	62/34 (35)	0.70	97/19 (16)	1.18 (0.50 to 2.75)	0.71
CRM asphalt workers§	38/9 (19)	32/10 (24)	0.99	23/13 (36)	0.30	30/8 (21)	1.89 (0.66 to 5.39)	0.24
Controls	59/41 (41)	67/31 (32)	0.13	47/26 (36)	0.24	86/12 (12)	Ref	Ref

*Lower airway symptoms included wheezing, chest tightness, shortness of breath and cough; upper airway symptoms included eye symptoms (redness/secretion/swelling) and nasal symptoms (runny nose/nasal congestion/sneezing), as well as nasal bleeding. Newly developed symptoms were the symptoms with onset during the working week, that is, no report of such symptoms on the preworking questionnaire, but were reported on the postworking questionnaire.

†P values were derived from asymptotic McNemar test with preworking symptoms as the comparison group.

‡OR and p values were derived from logistic regression adjusting for age, smoking history, cigarette pack-year and allergy.

§Four CRM asphalt workers did not report preworking symptoms. Nine CRM asphalt workers did not report postworking symptoms. Therefore, only 38 CRM asphalt workers were available to calculate newly developed symptoms.

CRM, crumb rubber modified; ref, reference.

To further investigate if a symptom is work-related or not in the longer term, we examined ‘symptoms with onset after beginning current job’, defined as a negative response to the question *‘Did you have the symptom before you began working in your current job?’*. The results showed that 26% of conventional and 20% of CRM asphalt workers, in contrast to only 10% among the controls, reported eye symptoms (redness/secretion/swelling) with onset after entering the current job (p=0.029). Similar patterns, although not significant, were also shown for wheezing and cough (table 4).

**Table 4:** Self-reported symptoms with onset after beginning current job*

	Conventional asphalt workers	CRM asphalt workers	All asphalt workers	Controls	P values†	P values‡
	(no/yes, %)	(no/yes, %)	(no/yes, %)	(no/yes, %)		
Wheezing	84/12 (13)	33/3 (8)	117/15 (11)	70/3 (4)	0.15	0.12
Shortness of breath	94/2 (2)	35/1 (3)	129/3 (2)	71/2 (3)	0.99	0.99
Cough	80/16 (17)	32/4 (11)	112/20 (15)	68/5 (7)	0.19	0.12
Eye symptoms (redness/secretion/swelling)	72/24 (25)	29/7 (19)	101/31 (23)	66/7 (10)	0.029	0.014
Nasal symptoms (runny nose/nasal congestion/sneezing)	70/26 (27)	29/7 (19)	99/33 (25)	59/14 (19)	0.46	0.39
Nasal bleeding	88/8 (8)	34/2 (6)	122/10 (8)	65/8 (11)	0.68	0.44

*Defined as a negative response to the question *‘Did you have the symptom before you began working in your current job?’* on the off-season questionnaire, to which 96 conventional asphalt workers, 36 CRM asphalt workers and 73 controls responded.

†P values were derived from χ^2^ test or Fisher’s exact test by comparing three occupational groups.

‡P values from χ^2^ test or Fisher’s exact test by comparing between all asphalt workers and controls.

### Lung function test

The CRM asphalt workers showed 4.6% higher preworking FVC (% predicted; 95% CI 0.5 to 8.7, p=0.03) and 3.8% higher FEV_1_ (% predicted; 95% CI −0.6 to 8.3, p=0.09) compared with the controls after adjustments. After 4 days of working, lung function parameters were decreased in CRM asphalt workers and controls, but not in conventional asphalt workers (table 5). No differences in lung function change (ΔFVC (% predicted) and ΔFEV_1_ (% predicted)) were noted among the three groups (p>0.38 for all). In the internal analysis with only asphalt workers involved, weak positive associations were suggested between years of asphalt work and ΔFVC (% predicted) (β=0.06, 95% CI −0.01 to 0.1, p=0.09), as well as ΔFEV_1_ (% predicted) (β=0.07, 95% CI −0.01 to 0.2, p=0.10).

**Table 5:** Preworking, postworking and absolute change from preworking to postworking of lung function parameters in three occupational groups*

Occupational groups	Preworking	Postworking	P values†	Mean (SD) of changes	β (95% CI)‡	P values‡
	Mean (SD)	Mean (SD)				
Parameters	FVC (% predicted)			ΔFVC (% predicted)		
Conventional asphalt workers	96.0 (11.8)	95.7 (11.3)	0.45	−0.3 (4.5)	0.6 (−0.7 to 1.9)	0.38
CRM asphalt workers	98.2 (9.8)	96.4 (9.9)	0.06	−1.2 (4.4)	−0.4 (−2.2 to 1.3)	0.64
Controls	92.9 (11.1)	92.0 (11.6)	0.08	−0.9 (4.9)	Ref	Ref

Parameters	FEV_1_ (% predicted)			ΔFEV_1_ (% predicted)		
Conventional asphalt workers	99.9 (13.3)	99.1 (12.5)	0.16	−0.7 (5.5)	0.6 (−0.9 to 2.0)	0.43
CRM asphalt workers	100.7 (12.8)	99.1 (11.8)	0.05	−1.6 (5.4)	−0.6 (−2.6 to 1.3)	0.51
Controls	97.6 (12.1)	96.2 (12.3)	0.008	−1.3 (4.7)	Ref	Ref

*Absolute change of FVC (% predicted) and FEV_1_ (% predicted) from preworking on Monday morning to postworking on Thursday afternoon; for example, ΔFVC (% predicted)=FVC (% predicted) (Thursday afternoon) − FVC (% predicted) (Monday morning). There were no missing data on lung function parameters.

†P values were derived from paired t-test.

‡β and p values were derived from general linear regression with smoking history, cigarette pack-year, allergy and pre-exposure FVC (% predicted) (or FEV_1_ (% predicted)) as adjustments.

CRM, crumb rubber modified; FEV_1_, forced expiratory volume in 1 s; FVC, forced vital capacity; ref, reference.

### Inflammation response

Preworking CRP was similar among the three groups, while preworking IL-8 was higher in CRM asphalt workers compared with the controls (β=8.80, 95% CI 4.79 to 12.8, p<0.001). After 4 days of working, CRP decreased in the controls but did not change in the two asphalt working groups. On the contrary, IL-8 decreased in both conventional and CRM asphalt workers but did not change in the controls (online supplementary table 2). No difference in the change in CRP or IL-8 was noted between the two asphalt groups and controls (online supplementary table 2). In the internal analysis including only asphalt workers, no association was found between years of asphalt work with either IL-8 (p=0.92) or CRP (p=0.34).

### Comparison between conventional and CRM asphalt paving in the repeated-measures analysis

In the repeated-measures analysis with 31 asphalt workers, 12 (40%) workers reported upper airway symptoms when paving with conventional asphalt, and 5 (17%) workers reported such symptoms when paving with CRM asphalt (p=0.25). Correspondingly, seven (23%) and five (17%) workers reported lower airway symptoms when paving with conventional and CRM asphalt, respectively (p=0.78). No difference in either lung function change or inflammatory cytokines change was noted between the two asphalt paving (p>0.17 for all, online supplementary table 3).

## Discussion

Our study showed a highly varied (up to two orders of magnitude) exposure to dust, which was similar in paving with CRM and conventional asphalt measured as particle mass under field conditions. However, in an experimental setting, the particle number emission of particles was higher for CRM than for conventional asphalt.^14^ Exposure to benzothiazole was higher among CRM asphalt workers than conventional asphalt workers, but no difference in airway symptoms, lung function or inflammation biomarkers change was discerned. Nitrosamines were unexpectedly found in conventional asphalt paving, indicating that an unknown source other than reused rubber tyres added in the asphalt may exist. Regarding health effects, a moderate decline in the lung function was found in CRM asphalt workers after paving for 4 days. Further, a slightly higher prevalence of lower airway symptoms in the working season compared with off-season was noted in conventional asphalt workers. These findings may indicate exposure-related effects on lower airway. Moreover, more reports of eye symptoms with onset after entering the current job in asphalt workers may suggest work-related irritation.

This study has several advantages. Instead of a cross-shift study with 1-day exposure,^2 20^ we performed a study with 4 days of exposure to represent a real working cycle in the asphalt industry in Sweden and to reduce day-to-day variation. We obtained full occupational and exposure histories to allow us to control possible confounders, such as potential exposure to other chemical agents. Repeated investigations of each participant offered the possibility to assess acute changes for both objective (changes in lung function and inflammatory cytokines) and subjective (symptoms) measures during the working week. The repeated-measures analysis has the advantage of workers serving as their own controls. However, despite the reasonable sample size in conventional asphalt workers and controls, fewer CRM asphalt workers and modest sample size in the repeated-measures analysis were a limitation with regard to discerning significant difference. Moreover, instead of choosing construction workers as the control group in previous studies,^21 22^ we chose employees working with lawn and garden maintenance due to similar working characteristics: outdoors, manual work and possible exposure to diesel exhaust. However, we unfortunately underestimated the potential effects from exposure to organic dust during mowing among the controls, which causes similar effects on airway. Besides, a higher prevalence of mite allergy among controls may imply some potential home exposures, which are difficult to assess. These might, to some extent, hold back a true difference between asphalt workers and the controls.

The personal exposure levels to respirable dust, total dust and total airborne PAHs were highly varied (up to two orders of magnitude) as may be expected in outdoor work, but most measurements were within the range of other studies involving open area paving workers.^23–25^ The levels were also lower than the current OELs in Sweden (5 mg/m^3^ for respirable dust and 10 mg/m^3^ for inhalable dust) and the threshold limit value (200 µg/m^3^) for PAHs set by the American Conference of Governmental Industrial Hygienists. However, since PAHs are non-threshold carcinogens, our observed levels may still be harmful. Moreover, the emitted particles are generally small (<1 µm^14^), yielding a large surface area per unit of mass, and the low effect levels reported for combustion products may thus be more relevant.^26^ Contrary to what we expected, two nitrosamines (N-nitroso-piperidine and N-nitroso-phenylamine) were found in both conventional and CRM asphalt workers, irrespective of whether conventional or CRM asphalt was used or what temperature was applied during paving. Since N-nitroso-dimethylamine and N-nitroso-morpholine are the most common detected nitrosamines in the rubber industry, including production of tyres,^27^ two detected nitrosamines in our study may suggest that the source was not only reused rubber tyres added in the asphalt mixture, but other additives. Given the fact that nitrosamines have shown potent carcinogenic effects in animal studies^28 29^ and are considered to be carcinogenic to human,^30 31^ it is important to identify and, if possible, eliminate the source of nitrosamines in asphalt paving. Our finding of higher exposure (almost six times) to benzothiazole among CRM asphalt workers than conventional asphalt workers was similar to other studies,^9 10^ indicating it may be useful as an indicator of CRM asphalt fumes exposure. However, no OEL for benzothiazole is available. Despite the finding of higher benzothiazole exposure during CRM asphalt paving, we did not find any differences in airway symptoms, lung function or inflammation response between paving with conventional and CRM asphalt, but it may still cause irritation when paving under unfavourable conditions.

The complex outdoor working environment resulted in highly varied exposure levels within the groups of conventional asphalt and CRM asphalt workers in our study. It was also likely to increase the variation in potential exposure-related symptoms, and therefore decrease the possibility of observing systematic differences between the groups. In our study, a slightly higher prevalence of lower airway symptoms in conventional asphalt workers during paving season compared with off-season, together with more reports of eye symptoms that started after entering the current job in asphalt workers than in the controls, may indicate a work-related airway irritation caused by exposure to asphalt fumes. Although the controls reported similar airway symptoms during working week, the reasons were different according to the open question in the questionnaire: the asphalt workers complained about the hot asphalt fumes, while the controls complained about mowing grass or weeds (organic dusts), which may cause similar irritative effects.

The generally higher lung function in the asphalt workers than in the controls, together with weak but positive associations between years of work and lung volumes among asphalt workers, could be due to healthy worker effect phenomenon.^32^ We found decreases in lung function in asphalt workers after 4 days of paving. Given the absence of diurnal improvement in lung function (ie, lung function is generally higher around 12:00 to 16:00 than in the early morning, around 06:00–08:00),^33^ the decreased lung function could be related to asphalt paving. However, a similar decline occurred in the controls and therefore no difference was found across the three groups. The cause of lung function decline in the controls is uncertain. One study found that exposure to grass pollen was associated with reduced lung function,^34^ which might be one reason. Another possible reason could be potential coexposure to diesel exhaust. However, we think it is less likely. In chamber studies with diesel exhaust,^35–37^ the transient airway irritation and lung function decline were detected when the exposure level was around 300 µg/m^3^. It is unlikely that the exposure level of diesel exhaust among the participants in our study can reach that level, especially in open areas.

Higher preworking IL-8 in CRM asphalt workers than in the controls could be a possible carryover effect from earlier exposure. The decrements of IL-8 in the asphalt workers after 4 days may indicate a shift from blood to the mucous membranes due to an inflammation process.^38^ However, these immune responses should be studied further to determine the mechanisms through which exposure to asphalt fumes can affect health.

## Conclusion

Asphalt workers are exposed to particles, airborne PAHs and nitrosamines at moderate, but highly varied levels. The source of nitrosamines in conventional asphalt needs to be identified. No acute airway irritation was found, but reduction in lung function after 4 days of paving was observed. Moreover, mild work-related symptoms (especially in the eyes) among asphalt workers were suggested. However, these findings need to be interpreted cautiously since the controls showed similar changes with regard to some effects. CRM asphalt paving was related to higher exposure to benzothiazole. No evidence of greater adverse health effects during CRM asphalt paving than conventional asphalt paving was found, which might be due to the smaller sample size in the repeated-measures analysis.

## References

[1] Burr G, Tepper A, Feng A, et al Health hazard evaluation report: crumb-rubber modified asphalt paving: occupational exposures and acute health effects. Cincinnati, OH: US Department of Health and Human Services, Centers for Disease Control and Prevention, National Institute for Occupational Safety & Health, 2002 (NIOSH HETA NO. 2001-0536-2864).

[2] Raulf-Heimsoth M, Pesch B, Schott K, et al Irritative effects of fumes and aerosols of bitumen on the airways: results of a cross-shift study. Arch Toxicol 2007;81:35–44. 10.1007/s00204-006-0115-z 16710697

[3] Sylvain D, Miller A Health Hazard Evaluation Report HETA 94-0219-2620. Boston, Massachusetts: Walsh Construction Company, 1997.

[4] WHO. Concise international chemical assessment document 59: asphalt (bitumen). Geneva: World Health Organization, 2004.

[5] Neghab M, Zare Derisi F, Hassanzadeh J Respiratory symptoms and lung functional impairments associated with occupational exposure to asphalt fumes. Int J Occup Environ Med 2015;6:113–21. 10.15171/ijoem.2015.473 25890605 PMC6977039

[6] Randem B, Exposure UB lung function and inflammation markers in asphalt workers. Norsk Epidemiologi 2009;19:229–34.

[7] Ellingsen DG, Ulvestad B, Andersson L, et al Pneumoproteins and inflammatory biomarkers in asphalt pavers. Biomarkers 2010;15:498–507. 10.3109/1354750X.2010.490305 20528258

[8] Swedish Criteria Group for Occupational Standards: Asphalt fumes around road paving, with focus on bitumen fume. Arbete och Hälsa 2011;45:2011.

[9] Burr G, Tepper A, Feng A, et al Crumb-rubber modified asphalt paving: occupational exposures and acute health effects. NIOSH Health Hazard Evaluation Report 2001-0536-2864. Cincinnati, OH, USA: National Institute for Occupational Safety and Health, 2001.

[10] Watts RR, Wallingford KM, Williams RW, et al Airborne exposures to PAH and PM2.5 particles for road paving workers applying conventional asphalt and crumb rubber modified asphalt. J Expo Anal Environ Epidemiol 1998;8:213–29. 9577752

[11] Stout D, Douglas P, Carlson D Stack Emissions With Asphalt Rubber: A Synthesis of Studies, CiteSeer. 2003 http://citeseerx.ist.psu.edu/viewdoc/download?doi=10.1.1.628.8696&rep=rep1&type=pdf (accessed 7 Apr 2018).

[12] Zanetti MC, Fiore S, Ruffino B, et al Assessment of gaseous emissions produced on site by bituminous mixtures containing crumb rubber. Constr Build Mater 2014;67:291–6. 10.1016/j.conbuildmat.2014.03.030

[13] Previa. Occupational health studies in pilot study 2006 and the rubber asphalt project 2007 – 2009: the Swedish Road Administration, 2010.

[14] Nilsson P, Bergendorf U, Tinnerberg H, et al Emissions into the air from bitumen and rubber bitumen – Implications for asphalt worker’s exposure, 2017. 10.1093/annweh/wxy053 29931293

[15] Xu Y, Lindh CH, Jönsson BAG, et al Occupational exposure to asphalt mixture during road paving is related to increased mitochondria DNA copy number: a cross-sectional study. Environ Health 2018;17:29 10.1186/s12940-018-0375-0 29587765 PMC5870390

[16] Jørgensen RB, Strandberg B, Sjaastad AK, et al Simulated restaurant cook exposure to emissions of PAHs, mutagenic aldehydes, and particles from frying bacon. J Occup Environ Hyg 2013;10:122–31. 10.1080/15459624.2012.755864 23343415

[17] Miller MR, Hankinson J, Brusasco V, et al Standardisation of spirometry. Eur Respir J 2005;26:319–38. 10.1183/09031936.05.00034805 16055882

[18] Berglund E, Birath G, Bjure J, et al Spirometric studies in normal subjects. I. Forced expirograms in subjects between 7 and 70 years of age. Acta Med Scand 1963;173:185-92. 13970718

[19] Federal Institute for Occupational Safety and Health. Technische regeln für gefahrstoffe, TRGS 552 N-nitrosamine, Bundesanstalt für Arbeitsschutz und Arbeitsmedizin (BAuA). Germany: Federal Institute for Occupational Safety and Health, 2007.

[20] Raulf-Heimsoth M, Pesch B, Rühl R, et al The Human Bitumen Study: executive summary. Arch Toxicol 2011;85(Suppl 1):3–9. 10.1007/s00204-011-0679-0 21369765

[21] Randem BG, Ulvestad B, Burstyn I, et al Respiratory symptoms and airflow limitation in asphalt workers. Occup Environ Med 2004;61:367–9. 10.1136/oem.2002.006114 15031397 PMC1740758

[22] Bergdahl IA, Järvholm B Cancer morbidity in Swedish asphalt workers. Am J Ind Med 2003;43:104–8. 10.1002/ajim.10157 12494427

[23] Butler MA, Burr G, Dankovic D, et al Hazard review: health effects of occupational exposure to asphalt: National Institute for Occupational Safety and Health, 2000.

[24] Norseth T, Waage J, Dale I Acute effects and exposure to organic compounds in road maintenance workers exposed to asphalt. Am J Ind Med 1991;20:737–44. 10.1002/ajim.4700200604 1805611

[25] Hicks JB Asphalt Industry Cross-Sectional Exposure Assessment Study. Appl Occup Environ Hyg 1995;10:840–8. 10.1080/1047322X.1995.10387699

[26] Lepeule J, Laden F, Dockery D, et al Chronic exposure to fine particles and mortality: an extended follow-up of the Harvard Six Cities study from 1974 to 2009. Environ Health Perspect 2012;120:965–70. 10.1289/ehp.1104660 22456598 PMC3404667

[27] de Vocht F, Burstyn I, Straif K, et al Occupational exposure to NDMA and NMor in the European rubber industry. J Environ Monit 2007;9:253–9. 10.1039/b615472g 17344951

[28] Swann PF, Magee PN Nitrosamine-induced carcinogenesis. The alkylation of N-7 of guanine of nucleic acids of the rat by diethylnitrosamine, N-ethyl-N-nitrosourea and ethyl methanesulphonate. Biochem J 1971;125:841–7. 10.1042/bj1250841 5145908 PMC1178189

[29] Magee PN, BARNES JM The production of malignant primary hepatic tumours in the rat by feeding dimethylnitrosamine. Br J Cancer 1956;10:114–22. 10.1038/bjc.1956.15 13342328 PMC2074083

[30] Straif K, Weiland SK, Bungers M, et al Exposure to high concentrations of nitrosamines and cancer mortality among a cohort of rubber workers. Occup Environ Med 2000;57:180–7. 10.1136/oem.57.3.180 10810100 PMC1739921

[31] Bartsch H, Montesano R Relevance of nitrosamines to human cancer. Carcinogenesis 1984;5:1381–93. 10.1093/carcin/5.11.1381 6386215

[32] Li CY, Sung FC A review of the healthy worker effect in occupational epidemiology. Occup Med 1999;49:225–9. 10.1093/occmed/49.4.225 10474913

[33] Spengler CM, Shea SA Endogenous circadian rhythm of pulmonary function in healthy humans. Am J Respir Crit Care Med 2000;162(3 Pt 1):1038–46. 10.1164/ajrccm.162.3.9911107 10988127

[34] Gruzieva O, Pershagen G, Wickman M, et al Exposure to grass pollen--but not birch pollen--affects lung function in Swedish children. Allergy 2015;70:1181–3. 10.1111/all.12653 26011717 PMC4744686

[35] Xu Y, Barregard L, Nielsen J, et al Effects of diesel exposure on lung function and inflammation biomarkers from airway and peripheral blood of healthy volunteers in a chamber study. Part Fibre Toxicol 2013;10:60 10.1186/1743-8977-10-60 24321138 PMC4029460

[36] Wierzbicka A, Nilsson PT, Rissler J, et al Detailed diesel exhaust characteristics including particle surface area and lung deposited dose for better understanding of health effects in human chamber exposure studies. Atmos Environ 2014;86:212–9. 10.1016/j.atmosenv.2013.11.025

[37] Giles LV, Carlsten C, Koehle MS The effect of pre-exercise diesel exhaust exposure on cycling performance and cardio-respiratory variables. Inhal Toxicol 2012;24:783–9. 10.3109/08958378.2012.717649 23033992

[38] Dierschke K, Isaxon C, Andersson UBK, et al Acute respiratory effects and biomarkers of inflammation due to welding-derived nanoparticle aggregates. Int Arch Occup Environ Health 2017;90:451–63. 10.1007/s00420-017-1209-z 28258373 PMC5486570

