# Supplementary material for: Exposure, respiratory symptoms, lung function and inflammation response of road-paving asphalt workers
Source: Occup Environ Med. 2018 May 30;75(7):494–500. doi: 10.1136/oemed-2017-104983 (PMC6035487; doi:10.1136/oemed-2017-104983)
Supplement: Supplementary data [file oemed-2017-104983supp001.pdf]

Supplementary Table 1: Self-reported symptoms from pre-, post-working and off-season questionnaires

		pre-working		post-working		off-season	
		(No/Yes, %)	p [a]	(No/Yes, %)	p [a]	(No/Yes, %)	p [a]
Wheeze	Conventional asphalt workers	109/7 (6%)		106/10 (9%)		89/7 (7%)	
	CRM asphalt workers	44/3 (6%)	0.28	41/1 (2%)	0.21	34/2 (6%)	0.93
	Controls	98/2 (2%)		95/3 (3%)		69/4 (5%)	
Chest tightness	Conventional asphalt workers	114/2 (2%)		113/3 (3%)		94/2 (2%)	
	CRM asphalt workers	46/1 (2%)	0.69	42/0 (0%)	0.85	35/1 (3%)	0.60
	Controls	96/4 (4%)		96/2 (2%)		69/4 (5%)	
Shortness of breath	Conventional asphalt workers	116/0 (0%)		115/1 (1%)		95/1 (1%)	
	CRM asphalt workers	47/0 (0%)	0.14	42/0 (0%)	0.99	36/0 (0%)	0.39
	Controls	97/3 (3%)		97/1 (1%)		70/3 (4%)	
Cough	Conventional asphalt workers	99/17 (15%)		101/15 (13%)		90/6 (6%)	
	CRM asphalt workers	42/5 (11%)	0.73	34/8 (19%)	0.54	33/3 (8%)	0.86
	Controls	89/11 (11%)		86/12 (12%)		69/4 (5%)	

Symptoms from eyes [b]	Conventional asphalt workers	98/18 (16%)		95/21 (18%)		90/6 (6%)	
	CRM asphalt workers	45/2 (4%)	0.017	39/3 (7%)	0.14	29/7 (19%)	0.086
	Controls	78/22 (22%)		87/11 (11%)		66/7 (10%)	
Symptoms from nose [b]	Conventional asphalt workers	80/36 (31%)		81/35 (30%)		70/26 (27%)	
	CRM asphalt workers	40/7 (15%)	0.081	34/8 (19%)	0.36	30/6 (17%)	0.38
	Controls	69/31 (31%)		69/29 (30%)		52/21 (29%)	
Nasal bleeding	Conventional asphalt workers	114/2 (2%)		113/3 (3%)		89/7 (7%)	
	CRM asphalt workers	46/1 (2%)	0.98	41/1 (2%)	0.99	33/3 (8%)	0.38
	Controls	98/2 (2%)		96/2 (2%)		71/2 (3%)	

a. P values were derived from χ^2 test or Fisher Exact test by testing difference across three occupational groups

b. Symptoms from eyes included eye redness, secretion and swelling. Symptoms from nose included runny nose, nasal congestion and sneezing.

Supplementary Table 2. Pre-, post-working, and absolute change from pre- to post-working of inflammatory cytokines (CRP and IL-8) in three occupational groups [a]

Occupational groups	Pre-working	Post-working	p [b]	Median (5%, 95%) of changes	β (95% CI) [c]	p [c]
	Median (5%, 95%)	Median (5%, 95%)				
Inflammation biomarkers	CRP			Δ CRP		
Conventional asphalt workers	1.0 (0.30, 5.1)	0.92 (0.30, 8.3)	0.65	0.001 (-1.5, 4.1)	0.41 (-0.20, 1.0)	0.19
CRM asphalt workers	1.1 (0.30, 4.7)	1.2 (0.30, 8.5)	0.42	0.001 (-0.90, 4.2)	0.47 (-0.39, 1.3)	0.28
Controls	1.0 (0.30, 9.7)	0.96 (0.30, 6.8)	0.04	-0.05 (-5.5, 1.6)	ref	ref
Inflammation biomarkers	IL-8			Δ IL-8		
Conventional asphalt workers	6.6 (0.34, 14)	5.9 (0.34, 14.8)	0.06	-0.16 (-4.8, 5.5)	-0.44 (-1.8, 0.90)	0.52
CRM asphalt workers	7.3 (4.2, 92)	6.2 (3.4, 15.1)	0.002	-0.79 (-83, 2.8)	-1.4 (-3.3, 0.49)	0.15
Controls	6.5 (3.8, 13)	7.0 (3.7, 12)	0.77	-0.06 (-4.3, 3.1))	ref	ref

a. Absolute change of CRP/IL-8 from pre-working on Monday morning to post-working on Thursday afternoon; e.g. Δ CRP = CRP (Thursday afternoon) - CRP (Monday morning). Three conventional asphalt workers, six CRM asphalt workers and four controls did not give the consent of blood sampling for biomarker test.

b. P values were derived from related-samples Wilcoxon Test

c. β and p values were derived from general linear regression with smoking history, cigarette pack-year, allergy and pre-exposure levels of biomarkers as adjustments.

Supplementary Table 3. Differences in the change of lung function and inflammation biomarkers between CRM asphalt paving and conventional asphalt paving in the repeated-measures analysis.

		β (95% CI) [a]	p [a]
Changes in lung function parameters	Δ FVC (%)	-1.5 (-3.7, 0.7)	0.18
	Δ FEV_1 (%)	-1.8 (-4.4, 0.8)	0.17
Changes in inflammatory biomarkers	Δ CRP	-0.28 (-2.11, 1.55)	0.76
	Δ IL-8	-0.26 (-1.95, 1.43)	0.75
	Δ SAA	-7.68 (-36.5, 21.1)	0.59

a. β and p values were derived from linear mixed model by comparing to conventional asphalt paving.
